# Some Facts on Incidence and Mortality of Cancer in Iran

**Published:** 2017-10

**Authors:** Mahdi MOHAMMADIAN, Hamid SALEHINIYA, Abdollah MOHAMMADIAN-HAFSHEJANI

**Affiliations:** 1. Dept. of Social Medicine, School of Medicine, Dezful University of Medical Sciences, Dezful, Iran; 2. Dept. of Epidemiology and Biostatistics, School of Public Health, Iran University of Medical Sciences, Tehran, Iran; 3. Dept. of Public Health, School of Health, Zabol University of Medical Sciences, Zabol, Iran; 4. Dept. of Epidemiology and Biostatistics, School of Public Health, Tehran University of Medical Sciences, Tehran, Iran

## Dear Editor-in-Chief

Cancer is one of the most common causes of death and health problems in the world (1). However, this illness diagnosed in the both sexes, all the ages’ group and races, the incidence and mortality of cancer are considerably different in various geographic areas. Based on WHO worldwide report’s on the cancer epidemic, the number of death for cancer in 2005 was 7.6 million which increased to 8.2 million in 2012 (2). Based on the first national cancer’ reports, the number of new cancer cases in Iran was 55855 in 2005. Cancer is the third common cause of death after cardiovascular disease and accidents in Iran (3) and leads to 50000 annual deaths (4).

**Fig. 1:**
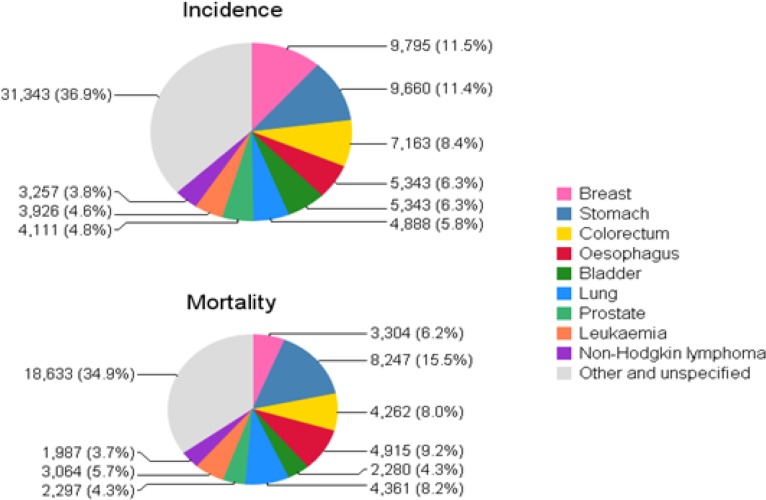
Cancers with The highest ASIR and ASMR in Iran in 2012

Based on the GLOBOCAN project data in Iran in 2012, 84822 newly diagnosed cancer observed (Age-Standardized Incidence Rates (ASIR) was 127.7 per 100000), developed in 44838 men (ASIR= 134.7) and 39991 women (ASIR=120.1). Cancers with the highest incident rate are including Breast, Stomach, Prostate, Colorectal and Esophagus cancer (Table 1 and Fig. 1) (5). Furthermore, in 2012 in Iranian population, 53350 death was reported because of cancer (Age-Standardized Mortality Rate (ASMR) was 81.9 per 100000), divided to 30115 men (ASMR= 90.4) and 23235 women (ASMR 72.7). The highest cause of mortality in this population was due to Stomach, Breast, Esophagus, Lung and Colorectal cancer, respectively (Table 1 and Fig. 1) (5). In Iranian, the risk (%) of getting cancer before age 75 is 13.1%, which is higher in men (9.4%) in comparison with women (7.8%) (2, 5).

**Table 1:** Cancers with the highest ASIR and ASMR in Iran in 2012

***Measures***	***Male***		***Female***		***Both sexes***	
**Incidence**	Cancer	Number(ASR)	Cancer	Number(ASR)	Cancer	Number(ASR)
	Stomach	6640(20.6)	Breast	9795(28.1)	Breast	9795(28.1)
	Bladder	4277(13.2)	Colorectal	3352(10.5)	Stomach	9660(15.3)
	Prostate	4111(12.6)	Stomach	3020(9.7)	Prostate	7163(11.1)
	Colorectal	3811(11.6)	Esophagus	2445(8)	Colorectal	5343(8.6)
	Lung	3307(10.3)	Lung	1637(4.8)	Esophagus	5343(8.4)
**Mortality**	Stomach	5665(17.3)	Breast	3304(9.9)	Stomach	8247(12.9)
	Lung	2950(09.1)	Stomach	2582(8.3)	Breast	3304(9.9)
	Esophagus	2662(08.3)	Esophagus	2253(7.4)	Esophagus	4915(7.8)
	Colorectal	2267(06.9)	Colorectal	1995(6.3)	Lung	4361(6.9)
	Prostate	2297(06.2)	Lung	1411(4.5)	Colorectal	4262(6.6)

Extracted from GLOBALCAN 2012

Due to some factors including; increased society members awareness about signs and symptoms of cancer, advance in diagnosis technic and equipment, improved cancer record system, increase in exposure to cancer risk factors, inactive lifestyle, changes in dietary habits and increasing proportion of elderly in the community, the incidence rate of cancer is increasing trend in Iranian population (6). In hence, health authorities and policy makers should plan for preventing or decreasing society exposure to common risk factors for cancer such as smoking, obesity, and sedentary lifestyle. Moreover, with consideration of increasing trend of cancer in Iran, planning and providing for advanced diagnostic tools and treatment centers and training experts can be an important priority for the national health system. To with expected increase in the incidence of the disease over the future years, the country's health system is able to offer health services at all levels of health care. Consequently, in addition to reducing the cancer incidence rate, case fatality rate and cause-specific mortality rate of cancer reduced.
